# Comprehensive Analysis of the Differential Expression and Prognostic Value of Histone Deacetylases in Glioma

**DOI:** 10.3389/fcell.2022.840759

**Published:** 2022-03-10

**Authors:** Jinwei Li, Xianlei Yan, Cong Liang, Hongmou Chen, Meimei Liu, Zhikang Wu, Jiemin Zheng, Junsun Dang, Xiaojin La, Quan Liu

**Affiliations:** ^1^ Department of Neurosurgery, The Fourth Affliated Hospital of Guangxi Medical University, Liuzhou, China; ^2^ College of Traditional Chinese Medicine, North China University of Science and Technology, Tangshan, China

**Keywords:** bioinformatics analysis, HDACs, immune infiltration, CGGA, Chinese Glioma Genome Atlas, biomarker, glioma

## Abstract

Gliomas are the most common and aggressive malignancies of the central nervous system. Histone deacetylases (HDACs) are important targets in cancer treatment. They regulate complex cellular mechanisms that influence tumor biology and immunogenicity. However, little is known about the function of HDACs in glioma. The Oncomine, Human Protein Atlas, Gene Expression Profiling Interactive Analysis, Broad Institute Cancer Cell Line Encyclopedia, Chinese Glioma Genome Atlas, OmicShare, cBioPortal, GeneMANIA, STRING, and TIMER databases were utilized to analyze the differential expression, prognostic value, and genetic alteration of HDAC and immune cell infiltration in patients with glioma. *HDAC1/2* were considerable upregulated whereas HDAC11 was significantly downregulated in cancer tissues. *HDAC1/2/3/4/5/7/8/11* were significantly correlated with the clinical glioma stage. *HDAC1/2/3/10* were strongly upregulated in 11 glioma cell lines. High *HDCA1/3/7* and low *HDAC4/5/11* mRNA levels were significantly associated with overall survival and disease-free survival in glioma. *HDAC1/2/3/4/5/7/9/10/11* are potential useful biomarkers for predicting the survival of patients with glioma. The functions of HDACs and 50 neighboring genes were primarily related to transcriptional dysregulation in cancers and the Notch, cGMP-PKG, and thyroid hormone signaling pathways. HDAC expression was significantly correlated with the infiltration of B cells, CD4^+^ T cells, CD8^+^ T cells, macrophages, neutrophils, and dendritic cells in glioma. Our study indicated that HDACs are putative precision therapy targets and prognostic biomarkers of survival in glioma patients.

## Introduction

Glioma is the most common tumor of the central nervous system (CNS). It accounts for 30% of all CNS tumors and 80% of all malignant brain tumors (Ostrom et al., 2019). Progress has been made in glioblastoma (GBM) treatment by combining maximal surgical resection with radiotherapy and concurrent and adjuvant temozolomide chemotherapy. Nevertheless, the two- and five-year survival rates are only 25 and 10%, respectively, (Stupp et al., 2005; Stupp et al., 2009). Tumor heterogeneity is a major challenge in precise glioma diagnosis and therapy (Neftel et al., 2019). Hence, molecular signatures for glioma are urgently required (Song et al., 2014).

Growing evidence suggests that epigenetic and genetic changes are pivotal in malignant disease onset and progression (Jones and Baylin, 2007). Histone deacetylases (HDACs) are important regulators of gene expression and control a broad array of physiological processes such as differentiation, apoptosis, survival, proliferation, and autophagy. They are also involved in cancer pathogenesis (Glozak and Seto, 2007; Wilson et al., 2008; Kang et al., 2014). There are four HDAC families and 18 individual HDACs. The zinc-dependent HDAC family comprises 11 isoforms divided into class I (HDAC1–3 and 8), class IIa (HDAC4, 5, 7, and 9), class IIb (HDAC6 and 10), and class IV (HDAC11) (Kashyap and Kakkar, 2020). Class III HDACs are homologous to yeast Sir2 and participate in transcriptional silencing. However, they have a deoxyhypusine synthase-like NAD/FAD-binding domain that is distinct from those of the other HDAC classes (Park et al., 2021). HDAC1 and two act mainly via nucleosome remodeling and deacetylase, switch independent 3, mitotic deacetylase, and corepressor of REST complexes. HDAC3 is recruited only by the nuclear receptor corepressor complex (Millard et al., 2017). HDAC6 regulates Hsp90, tau, and the cytoskeleton by interacting with tubulin and cortactin. It recognizes ubiquitinated proteins and induces aggregate formation (Hubbert et al., 2002; Kovacs et al., 2005; Dompierre et al., 2007; Zhang et al., 2007; Cohen et al., 2011; Simões-Pires et al., 2013). HDAC10 is a polyamine deacetylase (Ho et al., 2020), and HDAC11 has limited homology to class I and II enzymes (Li et al., 2015).

HDACs play important roles in many diseases. HDAC1 promotes medulloblastoma growth and affects cell cycle progression, microtubule dynamics, and DNA damage response (Abdelfattah et al., 2018). Preclinical studies suggest a role for HDAC1 in the epigenetic regulation of sarcoma tumorigenesis (Bennani-Baiti and Idriss, 2011). HDAC2 is highly expressed in sarcomas (Pacheco and Nielsen, 2012). HDAC3 is a target of the HDAC inhibitors approved by the US Food and Drug Administration to treat lymphoid malignancies (Stengel et al., 2019). HDAC4 overexpression has been observed in acute promyelocytic leukemia (Chauchereau et al., 2004), B cell lymphoma (Sandhu et al., 2012), and multiple myeloma (Kikuchi et al., 2015). HDAC6 is protective in tauopathy and suppresses aberrant tau accumulation. Chronic HDAC6 loss results in accelerated tau pathology, cognitive dysfunction, and reduced survival (Trzeciakiewicz et al., 2020). HDAC7 is a bona fide transcriptional repressor vital to B cell development (Azagra et al., 2016). HDAC8 modulates p53 activity and ensures long-term hematopoietic stem cell maintenance and survival under stress (Hua et al., 2017). HDAC5, HDAC7, and HDAC9 play protective roles during cardiac hypertrophy (Mckinsey, 2011). HDAC11 performs a key function in oncogene-induced, non-homeostatic hematopoiesis (11 deficiency disrupt, 2020).

Nevertheless, the mechanisms by which HDACs are inhibited or promoted in glioma development and progression are unclear. No comprehensive bioinformatics analysis has been conducted to identify the putative role of HDACs in glioma. In the present study, we mined numerous large databases to analyze HDAC expression, mutation, and function and immune infiltration and determine their potential oncogenic and prognostic value in glioma.

## Materials and Methods

### Oncomine Database

Oncomine (https://www.oncomine.org) contains 715 gene expression datasets and 86,733 samples. It is the largest oncogene chip database and incorporated data mining platform (§ et al., 2007). Transcriptional mRNA expression data for 11 HDACs in various cancers and their corresponding normal adjacent tissues were retrieved from Oncomine. The search content and threshold values were as follows: keywords, HDAC1–HDAC11; primary filter, cancer vs. normal; cancer type, absolute value of log2 │Fold Change│ > 1.5 and *p* < 0.05; and gene rank, 10%. *p*-values were calculated using the Student’s *t*-test.

### Human Protein Atlas

The Human Protein Atlas (https://www.proteinatlas.org) is an online tool including immunohistochemistry (IHC) expression data for protein distribution and expression in 20 cancer tissues, 48 human healthy tissues, 47 cell lines, and 12 blood cell types (Asplund et al., 2012). IHC images were used to compare HDAC protein expression levels among normal and cancer tissues. Localization of HDAC immunofluorescence expression in glioma cell lines was explored.

### Gene Expression Profiling Interactive Analysis

GEPIA (http://gepia.cancer-pku.cn/detail.php) is a new online interactive web server enabling users to examine RNA sequencing expression data for tumors, normal tissues, and samples in the Genotype-Tissue Expression projects and The Cancer Genome Atlas (TCGA). GEPIA is based on a criterion processing pipeline. It provides customizable functions such as tumor/normal differential expression analysis, profiling by cancer type or pathological stage, similar gene detection, and patient survival, correlation, and dimensionality reduction analyses (Tang et al., 2017). In the present study, differential gene expression analysis was used to compare tumors and normal tissues using GEPIA. The Student’s *t*-test was used to generate *p*-values for the expression analysis. A Kaplan–Meier curve for the patient survival analysis was also plotted.

### Broad Institute Cancer Cell Line Encyclopedia Database

The CCLE (https://www.broadinstitute.org/ccle) project conducts detailed genetic and pharmacologic characterizations of large panels of human cancer models. It develops integrated computational analyses linking distinct pharmacologic vulnerability to genomic patterns and provides public access to genomic data for the analysis and visualization of approximately 1,000 cell lines (Ghandi et al., 2019). The mRNA expression levels of 11 HDACs in multiple glioma cell lines were compared. The RNA expression dataset for 12 glioma cell lines was retrieved from CCLE and the HDAC expression levels were plotted in heat maps.

### Chinese Glioma Genome Atlas Database

CGGA (http://www.cgga.org.cn) is the largest glioma genome database in China. It provides multiple omics and matches clinical data for >2,000 primary and recurrent samples excised from Chinese cohorts (Zhao et al., 2020). In the present study, online tools and the mRNAseq_693 dataset were used to analyze the pathological stages associated with HDAC expression in patients with glioma.

### GeneMANIA Database and OmicShare

GeneMANIA (http://www.genemania.org) is a well maintained, user-friendly gene list analysis web interface used to derive hypotheses based on gene functions (Max et al., 2018). Here, GeneMANIA was utilized to construct a gene–gene interaction network of HDAC family members based on their physical interactions, predictions, co-expression levels, co-localizations, genetic interactions, drug-interactions-2020, transcriptional-factor-targets-2020, and so on. GeneMANIA was also used to evaluate HDAC functions. OmicShare (http://www.omicshare.com/tools) is a comprehensive platform for data processing and analysis, learning biological information, and sharing scientific research knowledge. Gene Ontology (GO) and Kyoto Encyclopedia of Genes and Genomes (KEGG) pathway enrichment analyses were performed using the free online data analysis platform OmicShare Tools (https://www.omicshare.com/tools).

### cBioPortal Databases

The cBioPortal (http://cbioportal.org) is a free asset that downloads large-scale cancer genomics datasets incorporating 245 cancer studies. The cBioPortal was used to explore genetic alterations in HDACs in glioma (Cerami et al., 2012). Here, the cBioPortal database was applied to explore 804 mutations in the brain lower-grade glioma (LGG; TCGA, and PanCancer Atlas) and glioblastoma multiforme (TCGA and Firehose Legacy) datasets for nerve center tumors. The distribution of HDAC mutations in glioma was calculated.

### STRING

STRING (https://string-db.org/) is a protein interaction website providing a comprehensive, objective global network. It presents its data using a unique set of computational predictions (Szklarczyk et al., 2019). A protein–protein interaction (PPI) network analysis was conducted through STRING to collect and integrate the expression of HDACs and their potential interactions.

### Tumor Immune Estimation Resource Analysis

The TIMER (https://cistrome.shinyapps.io/timer/) (Li et al., 2017) was used to evaluate the infiltration of CD8^+^ T cells, B cells, CD4^+^ T cells, macrophages, neutrophils, and dendritic cells in tumors and correlate them with HDAC expression in GBM and LGG. The survival module was used to establish prognostic relevance. *p* < 0.01 was considered statistically significant.

### Quantitative Real-Time Polymerase Chain Reaction of Tissues

Total RNA was extracted from tissue specimens using Animal RNA Isolation Kit (Invitrogen, Beyotime, Shanghai, China) according to the manufacturer’s instructions, and RNA was reversely transcribed into cDNA using Transcription First Strand cDNA synthesis kit (Beyotime, Shanghai, China). Quantitative real-time PCR (qRT-PCR) analyses were quantified with BeyoFast™ SYBR Green (Beyotime, Shanghai, China). The relative expression of HDACs were calculated based on the 2-ΔΔCt method with GADPH as an internal reference. qRT-PCR primers used in the present study were as follows:

HDAC1 forward primer, 5′- TCA​AGA​TGG​CCT​GAG​CAA​GG-3’; HDAC1 reverse primer, 5′-TGT​GCG​CTG​GTC​CCT​ATC​TA-3′; HDAC2 forward primer, 5′- TTC​CAA​GCC​CGA​CTG​TGA​GA-3′; HDAC2 reverse primer, 5′-ACC​TGT​TAG​AGC​CAG​TAA​GCA​C-3′; HDAC3 forward primer, 5′- GGC​CGA​TGC​TGA​AGA​GAG​AG-3′; HDAC3 reverse primer, 5′-GGG​GAT​ACC​CAG​TTC​AGA​CC-3′; HDAC4 forward primer, 5′- CCT​GTG​GCC​ACT​GCT​CTA​AA-3′; HDAC4 reverse primer, 5′-AAT​GCC​ATT​CTC​GGT​GCT​GA-3′; HDAC5 forward primer, 5′- CAG​GCT​GCT​GCC​ACT​CAA​GA-3′; HDAC5 reverse primer, 5′-CAC​AAT​GAT​GAA​GCC​CAG​AGG​G-3′; HDAC6 forward primer, 5′- AGC​TAG​TCC​TGT​GCC​GCT​A-3′; HDAC6 reverse primer, 5′-TGT​AGG​TAA​TGC​CGC​TGT​GG-3′; HDAC7 forward primer, 5′- ACC​TGC​GAG​TGG​GCC​AAA​G-3′; HDAC7 reverse primer, 5′-TAC​GGC​ACT​TCG​CTT​GCT​C-3′; HDAC8 forward primer, 5′- AAT​GAG​CCC​CAT​CGA​ATC​CAG-3′; HDAC8 reverse primer, 5′-GAT​ATC​CTC​CCT​CTT​TCC​CCC​TA-3′; HDAC9 forward primer, 5′- ACT​TGG​TTA​CCC​CAA​GGA​GC-3′; HDAC9 reverse primer, 5′-ATG​CAG​TGG​AGG​TCA​GAT​GC-3′; HDAC10 forward primer, 5′- TCA​CTG​GAC​AAG​CCT​CCA​C-3′; HDAC10 reverse primer, 5′-GGC​AAG​ATC​GTC​GTC​CTG​AA-3′; HDAC11 forward primer, 5′- TGC​TAA​AGA​GGC​CAT​CAG​GC′; HDAC10 reverse primer, 5′-TGA​GGA​TGG​AGT​CGG​CGA​TA-3′; GAPDH forward primer, 5′- CCG​CAT​CTT​CTT​GTG​CAG​TG′; GAPDH reverse primer, 5′- TCC​CGT​TGA​TGA​CCA​GCT​TC -3′

## Results

### Comparison of HDAC Expression Levels in Glioma and Normal Tissue Samples

To explore the potential prognostic and therapeutic values of various HDACs, we used the Oncomine and HPA databases to analyze their mRNA and protein expression levels in patients with glioma. TCGA statistics revealed that *HDAC1* expression was 3.131-fold higher (*p* = 3.08E-8) in ductal brain glioblastoma than in normal tissues (Table 1). Pomeroy (Pomeroy et al., 2002) has reported a 3.133-fold increase in *HDAC2* in desmoplastic medulloblastoma than in normal tissue samples (*p* = 5.47E-5). Gutmann has (Gutmann et al., 2002) observed a 2.483-fold increase in *HDAC3* in pilocytic astrocytoma than in normal samples (*p* = 0.021). Sun Brain Statistics (Sun et al., 2006) disclosed *HDAC6* overexpression in glioblastoma tissues compared with normal tissues and the fold change was 3.221 (*p* = 6.66E-13). The database search returned a 2.608-fold increase in *HDAC6* mRNA expression in anaplastic astrocytoma compared with normal tissues (*p* = 2.34E-6).

**TABLE 1:** Significant changes in histone deacetylase transcription between various types of glioma and normal tissues (Oncomine database).

	Database type	Tumor type	*p*-value	*t*-test	Fold change
HDAC1	TCGA Brain Statistics	Brain Glioblastoma vs. Normal	3.08E-8	14.612	3.131
HDAC2	Pomeroy Brain Statistics (Ostrom et al., 2019)	Desmoplastic Medulloblastoma vs. Normal	5.47E-5	6.188	3.133
HDAC3	Gutmann Brain Statistics (Stupp et al., 2009)	Pilocytic Astrocytoma vs. Normal	0.021	3.649	2.483
HDAC6	Sun Brain Statistics (Stupp et al., 2005)	Glioblastoma vs. Normal	6.66E-13	8.831	3.221
	Sun Brain Statistics	Anaplastic Astrocytoma vs. Normal	2.34E-6	5.48	2.608
Differences in transcriptional expression were compared by Students’ t-test. Cut-off of *p*-value and fold-change were as follows: *p*-value: 0.01,fold-change: 1.5, gene rank: 10%					

**FIGURE 1:**
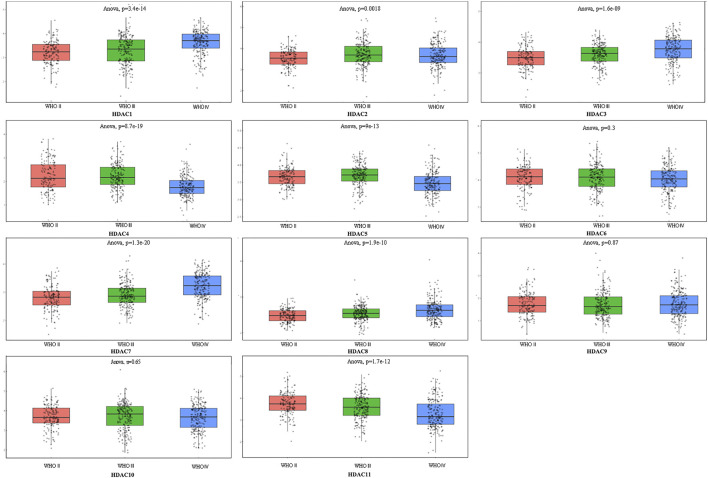
Comprehensive analysis of prognosis and histone deacetylase expression in glioma. HDAC1/2/3/4/5/7/8/11 expression levels were correlated with pathological stage of glioma patients (*p* < 0.05).

**FIGURE 2:**
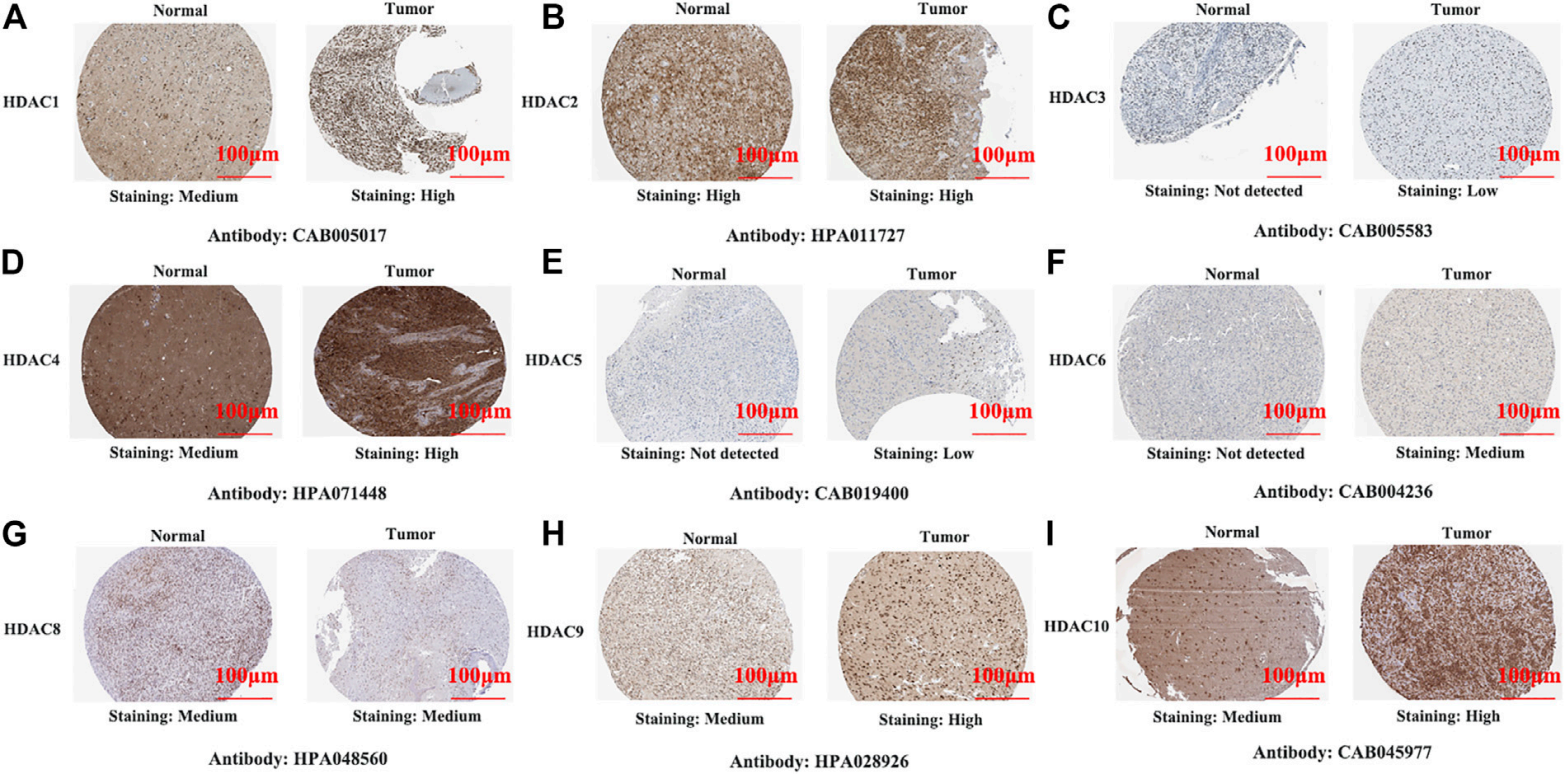
Representative immunohistochemistry images of histone deacetylase family members in glioma and normal tissues (Human Protein Atlas Database). All tissues were observed at ×100 magnification.

We also explored protein-level HDAC expression using the Human Protein Atlas database. Low and medium HDAC1/4/8/9/10 protein expression levels were determined for normal tissues, and high HDAC1/4/8/9/10 protein expression levels were found in glioma tissues (Figures 1A,D,G,H). Figures 2C,E,F show that HDAC3/5/6 proteins are not expressed in normal gastric tissues but are detected at low and medium levels in glioma tissues.

### Relationships Between HDAC mRNA Levels and Clinicopathological Parameters of Patients with Glioma

**FIGURE 3:**
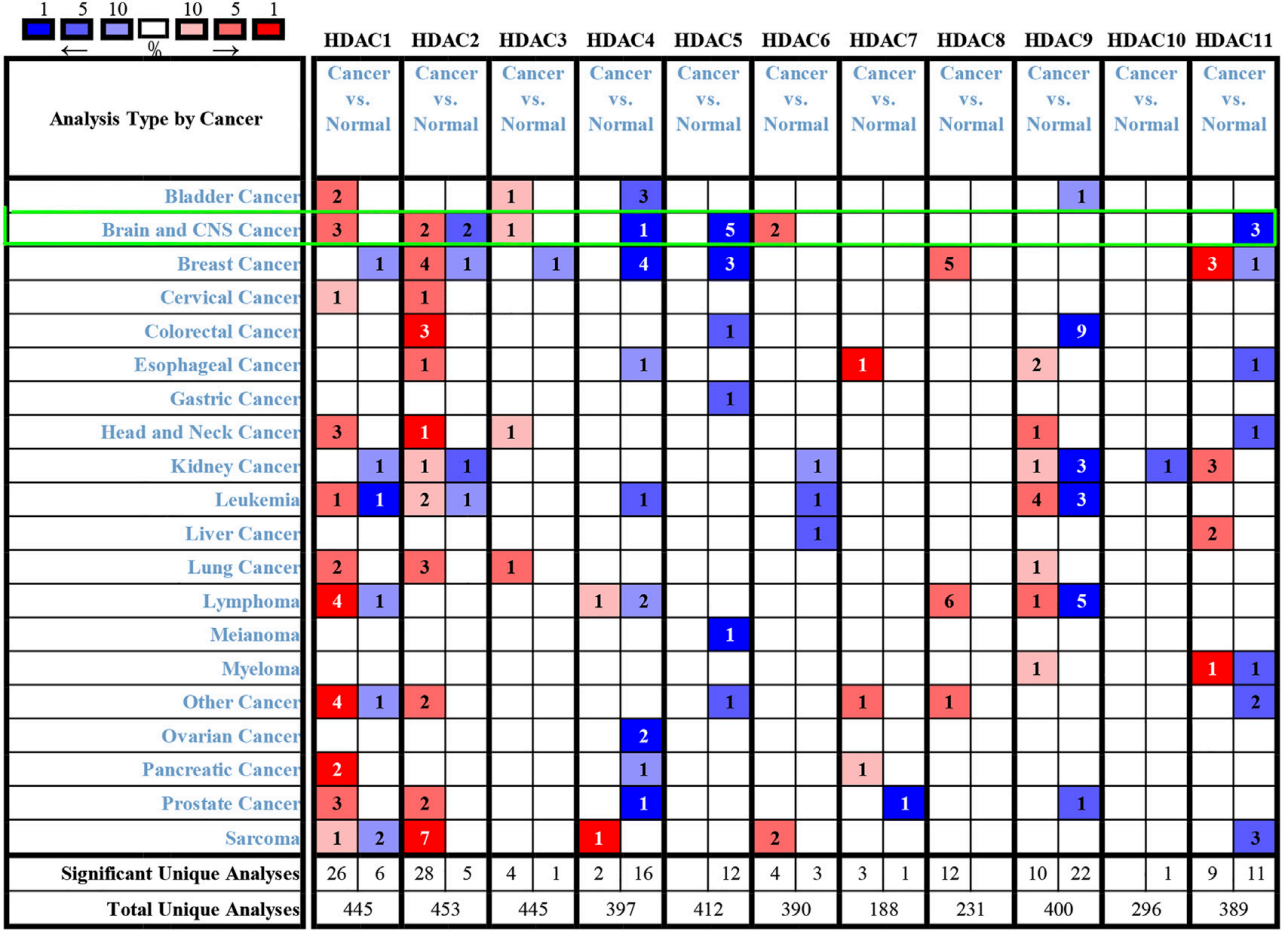
Histone deacetylase transcription levels in various cancers (Oncomine). Numbers in colored cells show quantities of datasets with statistically significant target gene mRNA upregulation (red) or downregulation (blue). The following criteria were used: *p*-value, 0.01; fold change, two; gene rank, 10%; data type, mRNA; analysis type, cancer vs. normal tissue. CNS: central nervous system.

**FIGURE 4:**
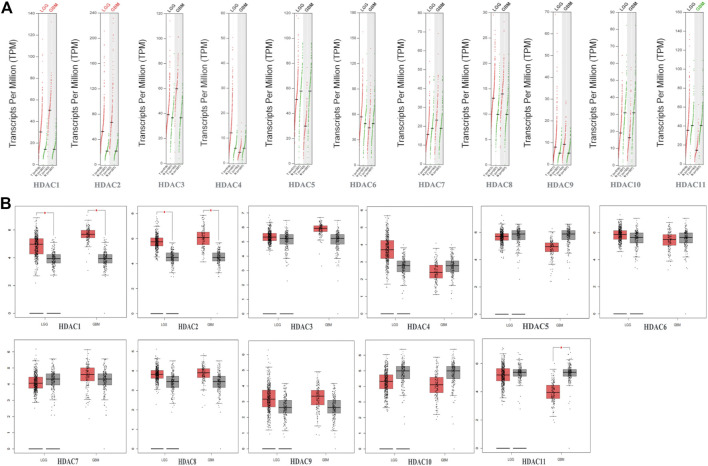
Gene expression profiling interactive analysis data for histone deacetylase (HDAC) mRNA expression in glioma including glioblastoma (GBM) and lower-grade glioma (LGG) compared with NB tissue **(A,B)** Scatter diagram and Box plot showing that HDAC1/2 expression levels in GBM (*n* = 163) and LGG (*n* = 518) tumor tissues were higher than those in normal tissues (*n* = 207). HDAC11 expression levels were lower in GBM tumor tissues than in normal tissues (*p* < 0.05).

**FIGURE 5:**
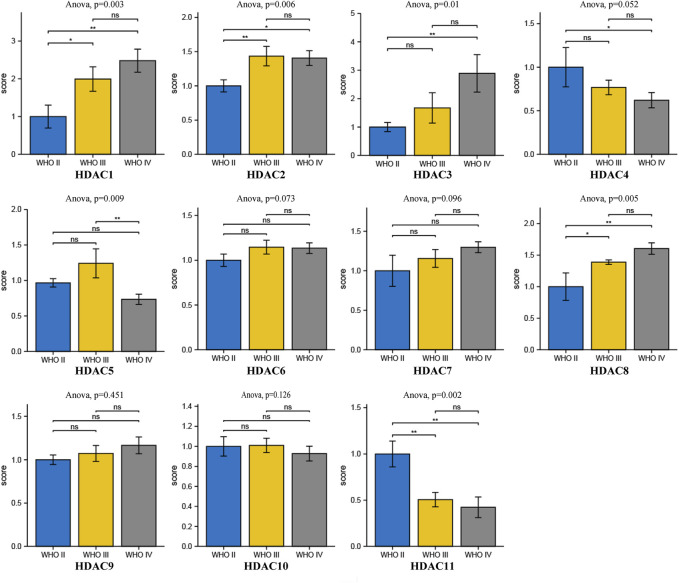
Comprehensive analysis of prognosis and histone deacetylase expression in glioma. HDAC *HDAC1/2/3/4/5/8/11* expression levels were correlated with pathological stage of glioma patients (*p* < 0.05).

To explore distinct HDAC expression in patients with glioma, HDAC mRNA expression was analyzed using the Oncomine database (Figure 3). The Oncomine data revealed that compared with normal tissues, the *HDAC1/2/3/6* transcriptional levels were significantly elevated and those of *HDAC2/4/5/11* were significantly decreased in CNS cancer tissues. The GEPIA dataset was used to compare HDAC mRNA expression between glioma and normal tissues. *HDAC1* and *HDAC2* were relatively upregulated and HDAC11 was downregulated in LGG and GBM tumor tissues (Figure 4). We also compared HDAC expression levels across glioma tumor stages. *HDAC1/2/3/4/5/7/8/11* expression significantly varied across glioma stages, whereas *HDAC6/9/10* expression did not (Figure 1). Therefore, qRT-PCR was performed to verify the expression of HDAC family gene mRNA in glioma tumor tissues. *HDAC1/2/3/4/5/8/11* expression levels were correlated with pathological stage of glioma patients (*p* < 0.05) (Figure 5).

### HDACs mRNA Expression and Immunofluorescence in Glioma Cells

**FIGURE 6:**
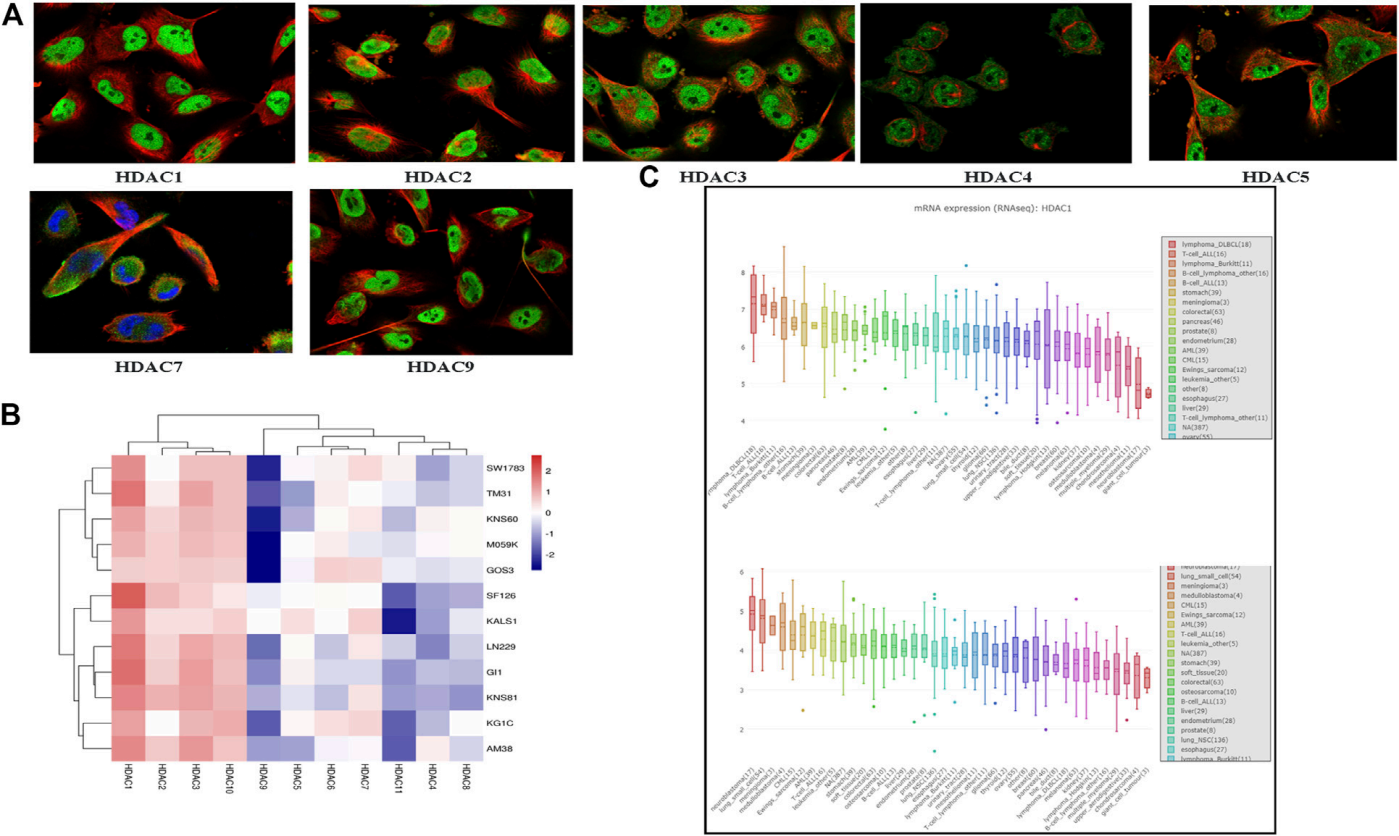
Cell line histone deacetylase (HDCA) mRNA expression levels (Cancer Cell Line Encyclopedia) **(A)** HDAC1/2/3/4/5/7/9 immunofluorescence was expressed in glioma U-251 MG cell nuclei **(B)** HDAC1/2/3/10 mRNA was highly expressed in 12 glioma cell lines **(C)** Box plot was sorted and colored according to average distribution of HDAC1 and HDAC2 expression in various tumor cell lines. Immunofluorescence analysis of HDAC1/2/3/4/5/7/9 in U-251 MG.

We compared HDAC expression levels among the foregoing databases and used CCLE to explore HDAC1/2 expression levels in glioma cell lines. All HDACs were strongly upregulated in glioma cell lines (Figure 6C). We also found that *HDAC1/2/3/10* were highly expressed in 11 glioma cell lines from the CCLE database, namely, SW1783, TM31, KNS60, M059K, GOS3, SF126, KALS1, LN229, GI1, KNS81, KG1C, and AM38 (Figure 6B). We searched the cell atlas of the HDAC family in glioma and observed immunofluorescence of *HDAC1/2/3/4/5/7/9* (Figure 6A). In the human protein map, most HDAC proteins were localized to and upregulated in human glioma cell nuclei.

### Prognostic Significance of HDACs in Patients with Glioma

**FIGURE 7:**
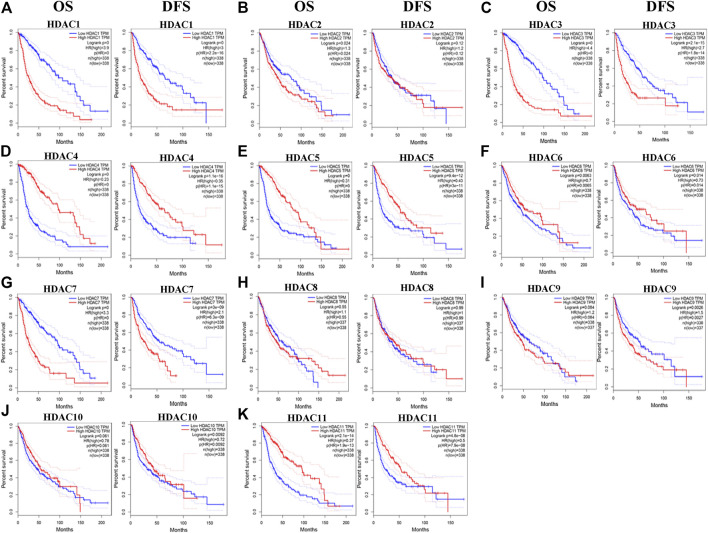
Prognostic value of histone deacetylase mRNA expression in patients with glioma (Gene expression profiling interactive analysis). *p < 0.05. HR, hazard ratio; OS, overall survival; DFS, disease-free survival.

We evaluated the prognostic significance of HDACs in patients with glioma using the GEPIA and CGGA databases. A log-rank test analysis revealed that high *HDCA1/3/7* and low *HDAC4/5/11* mRNA levels were significantly associated with overall survival (OS) and disease-free survival (DFS) (*p* < 0.05) (Figure 7). *HDAC9* mRNA downregulation and *HDAC10* mRNA upregulation had prognostic value in DFS, whereas *HDAC2* mRNA upregulation had prognostic value in OS (*p* < 0.05). The GEPIA database showed that *HDAC1/2/3/4/5/7/9/10/11* mRNA expression levels were significantly associated with glioma patient prognosis and could, therefore, be exploited as biomarkers for the prediction of glioma patient survival.

### Genetic HDAC Alteration Analysis in Glioma Patients

**FIGURE 8:**
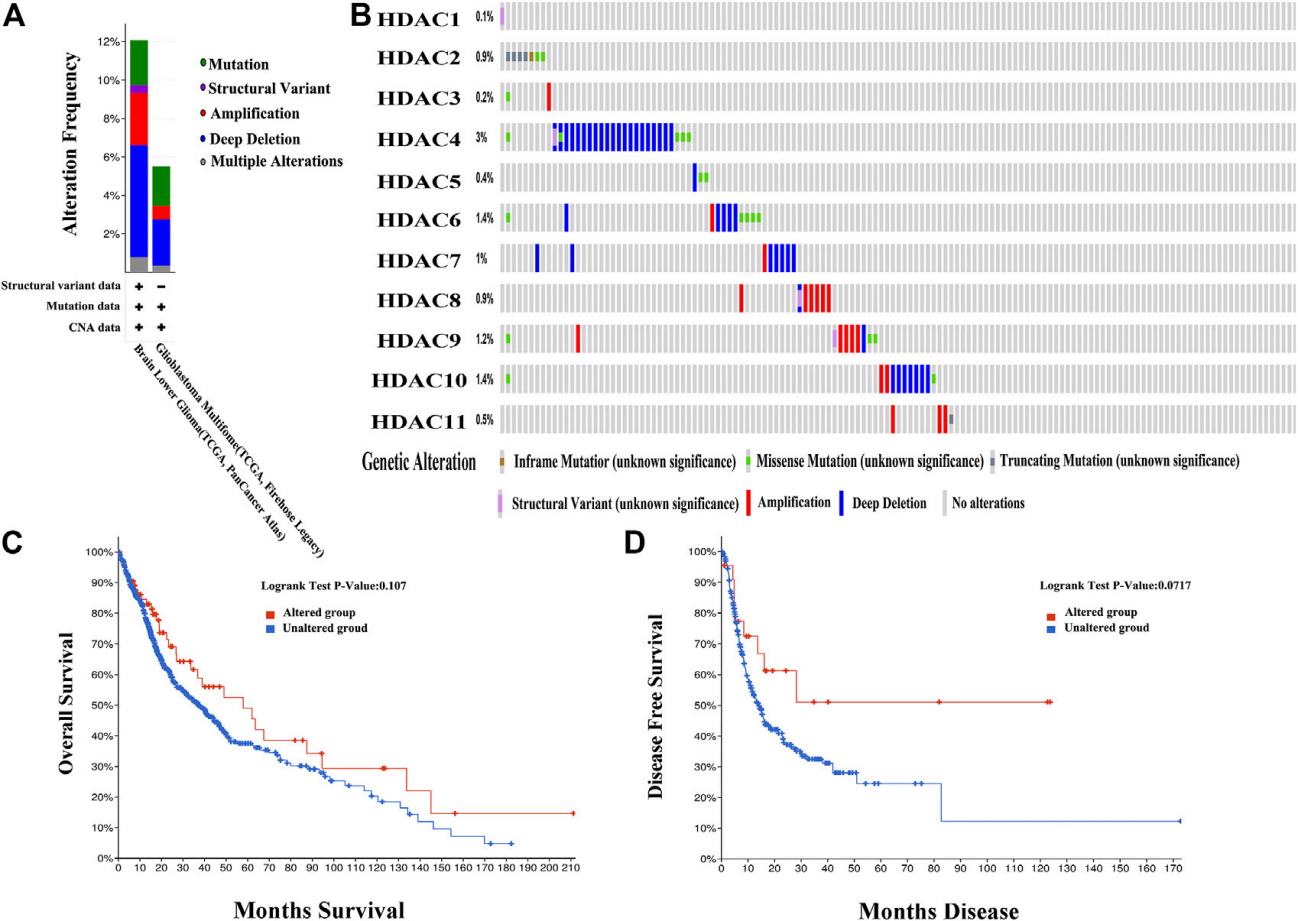
Genetic histone deacetylase (HDAC) alteration analysis in patients with OS (cBioPortal) **(A)** HDAC alterations **(B)** OncoPrint tab summary of query of HDAC alterations. Kaplan–Meier plots comparing **(C)** overall survival (OS) and **(D)** disease-free survival (DFS) in patients with and without HDAC gene alterations.

Genetic alterations in the HDACs of glioma patients were examined using TCGA database and the cBioPortal online tool. HDACs were altered in 804 samples from 1,118 glioma patients in TCGA databases of brain LGG and GBM. The alteration rates were 12.05% (62/514) and 5.52% (16/290), respectively. Deep deletion accounted for most of the observed changes (Figure 8A). Figure 8B shows that genetic HDAC alterations occurred in 78 (10%) of queried samples and that the alteration rates in the individual sequence were in the range of 0.1–3%. HDAC4, HDAC6, and HDAC10 were ranked as the top three of the eleven HDAC members and their deep deletion rates were 3, 1.4, and 1.4%, respectively, (Figure 8B). The “Survival” tab, the Kaplan–Meier plot, and the log-rank test plotted survival curves indicated that cases with or without HDAC alterations had no correlation with OS or DFS (Figures 8C,D).

### Predicted HDAC and HDAC-Related Neighboring Gene Functions and Pathways in Patients with Glioma

**FIGURE 9:**
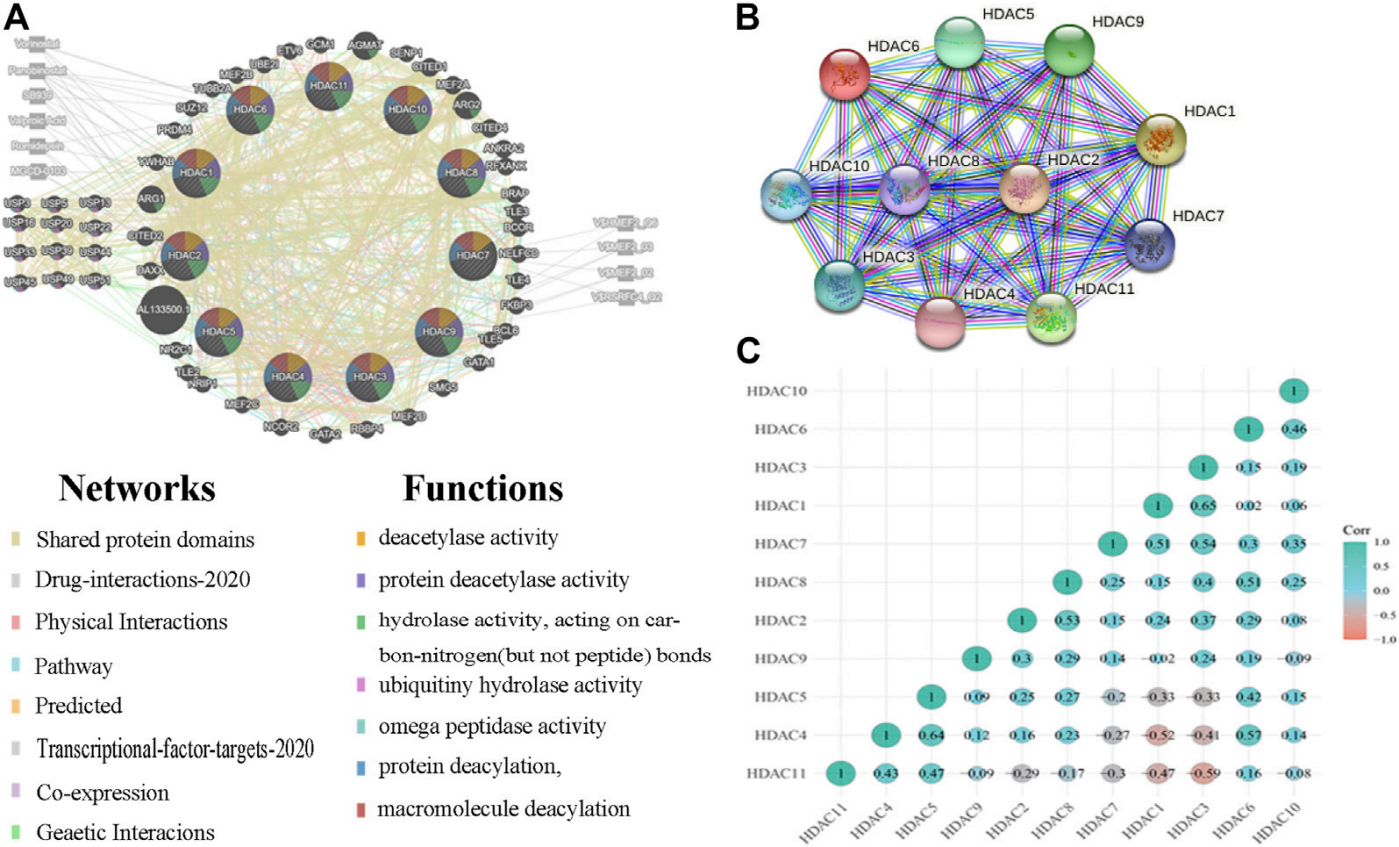
Protein–protein interactions (PPI) among histone deacetylases (HDACs) **(A)** Network of HDACs and their 50 neighboring genes was constructed using GeneMANIA **(B)** PPI network of various HDACs **(C)** Pearson correlation coefficients among HDACs.

We analyzed 50 neighboring genes significantly related to HDACs and used GeneMANIA to construct an integrated network. Figure 8A shows that according to the weight analysis, HDACs were associated with ubiquitinase-related genes, including USP3/5/13/16/20/22/33/39/44/49/45/51. Gene-related drugs acting on HDACs included panobinostat, vorinostat, romidepsin, SB939, valproic acid, and MGCD-0103. The transcription factors HMEF2_Q6, RSRFC4_Q2, MEF2_02, and MEF2_03 were associated with HDAC-related genes. The LGG and GBM mRNA data in TCGA and the Spearman’s correlation analyses were used to calculate correlations among HDAC family members. The STRING database was used to build a PPI network for the HDAC family (Figures 9A,B). There were significant positive correlations between the following HDACs: HDAC1 with HDAC3; HDAC2 with HDAC3 and HDAC8; HDAC4 with HDAC5 and HDAC6; HDAC5 with HDAC6; HDAC6 with HDAC10; HDAC7 with HDAC1, HDAC3, HDAC6, and HDAC10; HDAC8 with HDAC3 and HDAC6; HDAC9 with HDAC2; and HDAC11 with HDAC4 and HDAC5. There were also significant negative correlations between the following HDACs: HDAC4 with HDAC1 and HDAC3; and HDAC5 with HDAC1 and HDAC3 (Figure 9C).

**FIGURE 10:**
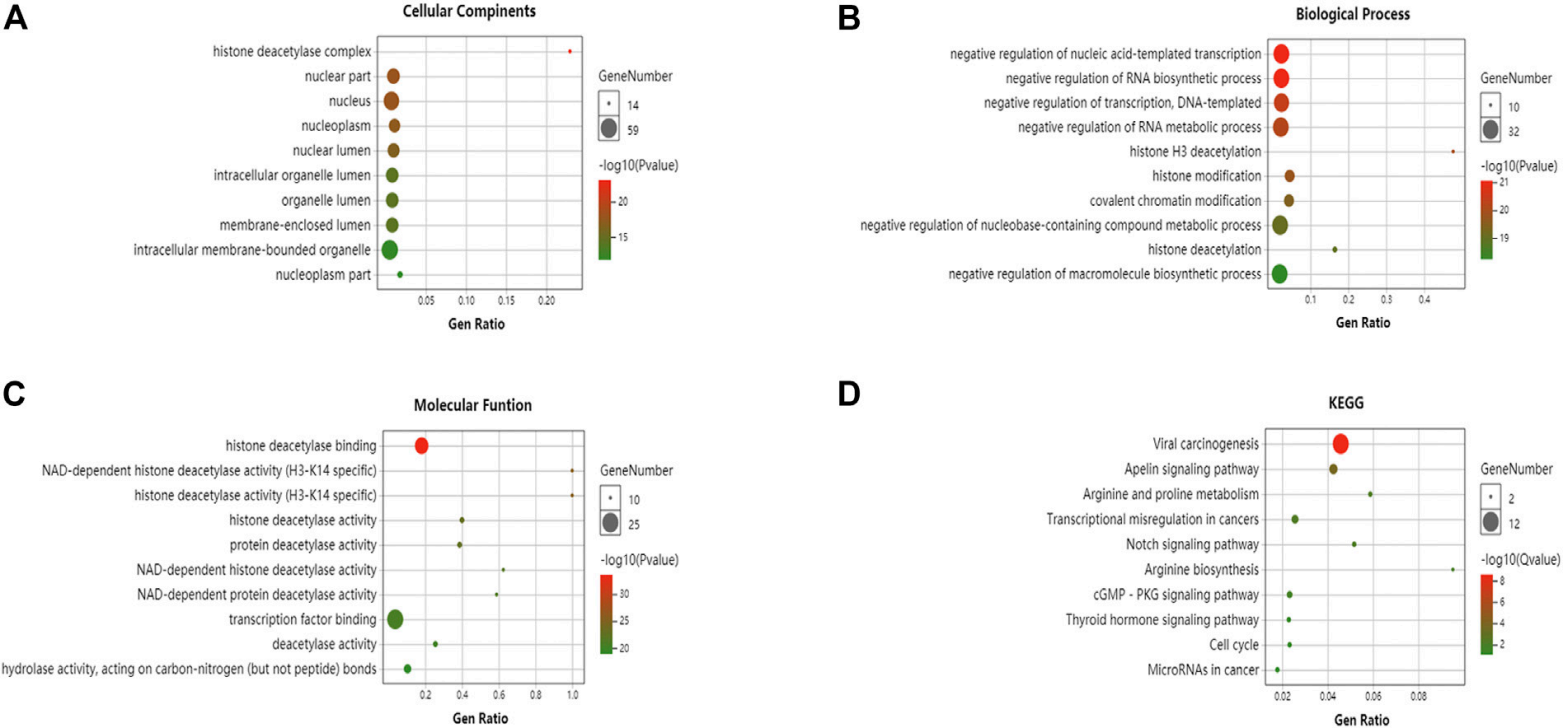
Gene ontology (GO) enrichment and Kyoto Encyclopedia of Genes and Genomes (KEGG) pathway analyses of HDACs and their interactions (OmicShare). GO enrichment analysis of target genes based on **(A)** cellular component, **(B)** biological process, and **(C)** molecular function **(D)** KEGG pathway enrichment analysis of target genes.

GO enrichment and KEGG pathway analyses of the HDACs and 50 neighboring genes were performed using OmicShare. The top ten most highly enriched GO items were identified (Figures 10A–C). The GO term analysis showed that differentially expressed genes correlated with the HDACs were localized mainly with the histone deacetylase complex, nuclear part, nucleus, nucleoplasm, nuclear lumen, and intracellular organelle lumen; HDACs negatively regulate RNA biosynthesis and transcription, DNA templates, RNA metabolism, histone H3 deacetylation, and histone modification at these sites. HDACs have histone deacetylase-binding, H3-K14-specific NAD-dependent histone deacetylase and histone deacetylase activity, protein deacetylase activity, NAD-dependent histone deacetylase activity, and so on. The KEGG pathway analysis disclosed viral carcinogenesis, apelin signaling, arginine and proline metabolism, transcriptional misregulation in cancer, Notch signaling, and cGMP-PKG signaling. The thyroid hormone signaling pathway and arginine biosynthesis were significantly associated with glioma tumorigenesis and progression (Figure 10D).

### HDAC Expression and Immune Infiltration in Glioma

**FIGURE 11:**
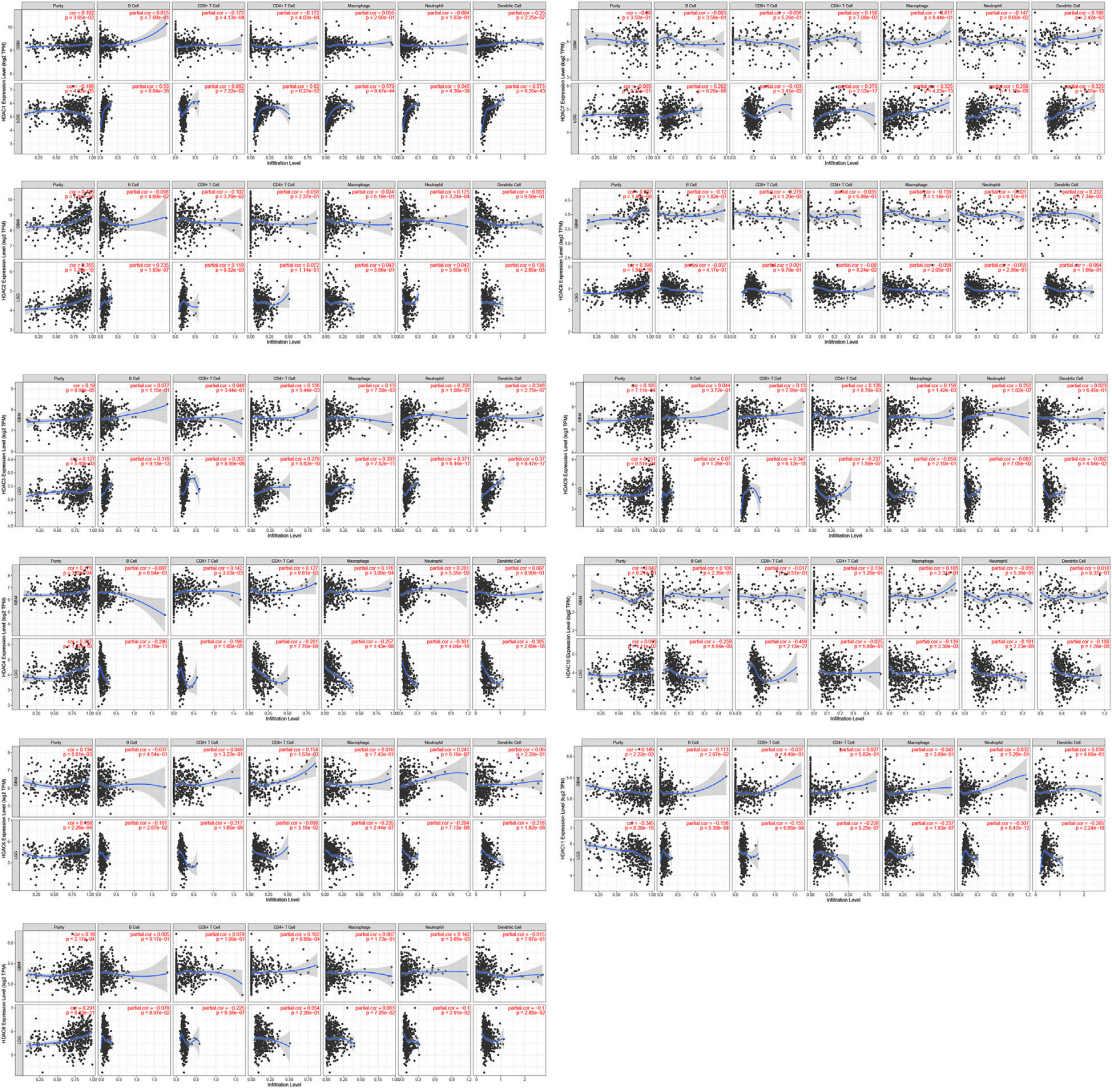
Correlations among differentially expressed HDACs and immune cell infiltration (TIMER). Correlations between immune cell abundance and HDAC1–11 expression.

The TIMER database was used to investigate associations among the HDACs family members and immune cell infiltration. The immune cell count is correlated with cancer cell proliferation and progression (Figure 11). *HDAC1* expression in LGG was positively correlated with the infiltration of B cells (*p* < 0.001), CD4^+^ T cells (*p* < 0.01), macrophages (*p* < 0.01), neutrophils (*p* < 0.001), and dendritic cells (*p* < 0.01). *HDAC2/8* expression levels in LGG and GBM were positively correlated with purity infiltration (*p* < 0.01).

*HDAC3* expression in LGG was positively correlated with the infiltration of B cells (*p* < 0.01), neutrophils (*p* < 0.01), and dendritic cells (*p* < 0.01). *HDAC7* expression in LGG was positively correlated with the infiltration of CD4^+^ T cells (*p* < 0.01), macrophages (*p* < 0.01), and dendritic cells (*p* < 0.001). *HDAC9* expression in LGG was positively correlated with CD8^+^ T cell infiltration (*p* < 0.001). *HDAC11* expression in LGG was positively correlated with purity infiltration (*p* < 0.01) and negatively correlated with neutrophil infiltration (*p* < 0.01). A Cox proportional hazard model of HDACs and clinical factors in glioma was also evaluated. *HDAC3/4* expression, patient age, and CD4^+^ T cell density were significantly associated with clinical outcomes in GBM (Table 2). *HDAC1* expression and patient age were significantly associated with the clinical prognosis of LGG (Table 3).

**TABLE 2:** Multivariate survival model analysis based on TIMER online tool (glioblastoma).

	Coef	HR	95%CI_l	95%CI_u	*p*.value	Sig
Age	0.033	1.033	1.007	1.060	0.012	*
Gender male	0.414	1.512	0.881	2.596	0.133	—
Race Black	−0.156	0.855	0.150	4.892	0.860	—
Race White	−0.510	0.600	0.150	2.406	0.471	—
Purity	−0.850	0.427	0.072	2.530	0.349	—
B_cells	−0.376	0.686	0.082	5.725	0.728	—
CD8+Tcell	0.114	1.121	0.271	4.632	0.875	—
CD4+Tcell	4.274	71.838	3.371	1,530.896	0.006	**
Macrophage	2.002	7.405	0.507	108.057	0.143	—
Neutrophil	−1.933	0.145	0.005	4.585	0.273	—
Dendritic	0.932	2.539	0.842	7.656	0.098	
HDAC1	−0.741	0.476	0.206	1.101	0.083	—
HDAC2	0.242	1.274	0.721	2.251	0.405	—
HDAC3	1.272	3.569	1.322	9.633	0.012	*
HDAC4	−1.340	0.262	0.111	0.618	0.002	**
HDAC5	0.469	1.598	0.650	3.925	0.307	—
HDAC6	0.763	2.144	0.814	5.650	0.123	
HDAC7	−0.293	0.746	0.400	1.390	0.356	—
HDAC8	−0.211	0.810	0.276	2.372	0.700	—
HDAC9	0.132	1.141	0.792	1.644	0.479	—
HDAC10	0.017	1.017	0.576	1.796	0.954	—
HDAC11	0.236	1.267	0.747	2.147	0.380	—

Coef: coefficient, HR: hazard ratio, CI: confidence interval, sig: significance. *p < 0.05.

**TABLE 3:** Multivariate survival model analysis based on TIMER online tool (lower-grade glioma).

	Cof	HR	95%CI_l	95%CI_u	*p*.value	Sig
Age	0.059	1.061	1.043	1.079	0.000	***
Gender male	0.245	1.277	0.827	1.973	0.270	—
Race Black	16.758	18,961,194.043	0	Inf	0.995	—
Race White	17.125	27,383,645.009	0	Inf	0.994	—
Purity	−0.225	0.799	0.269	2.372	0.686	—
B_cell	0.932	2.540	0.001	4,482.746	0.807	—
CD8_Tcell	1.699	5.466	0.003	9,731.339	0.656	—
CD4_Tcel	−2.442	0.087	0.000	1,089.174	0.612	—
Macrophage	4.756	116.240	0.842	16,038.885	0.059	—
Neutrophil	−6.479	0.002	0.000	11.959	0.156	—
Dendritic	1.393	4.025	0.036	454.222	0.564	—
HDAC1	0.815	2.260	1.408	3.627	0.001	**
HDAC2	0.209	1.233	0.715	2.126	0.451	—
HDAC3	0.148	1.160	0.510	2.636	0.723	—
HDAC4	−0.098	0.907	0.597	1.378	0.647	—
HDAC5	−0.342	0.710	0.377	1.338	0.290	—
HDAC6	−0.353	0.703	0.312	1.583	0.395	—
HDAC7	0.215	1.240	0.618	2.489	0.544	—
HDAC8	0.521	1.685	0.732	3.878	0.220	—
HDAC9	0.113	1.119	0.833	1.504	0.455	—
HDAC10	0.245	1.278	0.833	1.959	0.261	—
HDAC11	0.265	1.304	0.833	2.041	0.245	—

Coef: coefficient, HR: hazard ratio, CI: confidence interval, sig: significance. *p < 0.05.

## Discussion

Epigenetic modifications are heritable, reversible genetic changes that do not involve DNA mutation. HDACs are epigenetic regulators that remove the acetyl moieties from acetylated lysine residues of post-translationally modified histone proteins that form the core of the chromatin network. This mechanism compresses chromatin, compromises DNA accessibility, and represses transcription and gene silencing (Sengupta and Seto, 2004). However, distinct roles of each HDAC family member in glioma remain to be clarified. In the present study, we analyzed HDAC expression, mutation, prognostic value, functional enrichment, and immune cell infiltration in patients with glioma. To this end, we consulted various public databases including Oncomine, Human Protein Atlas, GEPIA, cBioPortal, CCLE, GeneMANIA, String, and CGGA. GO enrichment and KEGG pathway were analyzed with OmicShare Tools.

Previous studies have evaluated the roles of certain HDAC family members in glioma (Lodrini et al., 2013; Wang et al., 2017; Lo Cascio et al., 2019; Song et al., 2020). A previous study has indicated that the NFAT2-HDAC1 pathway may maintain the malignant phenotype and promote mesenchymal transition in glioma stem-like cells (GSCs). Hence, GSCs are potential molecular targets for GBM therapy (Song et al., 2020). HDAC1 ablation significantly decreased stemness and proliferation in patient-derived GSCs in a p53-dependent manner. By contrast, it had only minimal impact on normal human neural stem cells and astrocytes (Lo Cascio et al., 2019). HDAC1 knockdown inhibited the epithelial–mesenchymal transition transcription factors TWIST1 and SNAIL, downregulated the mesenchymal marker matrix metalloprotein-9, and upregulated the epithelial marker E-cadherin 34. Hence, HDAC1 may contribute to epithelial–mesenchymal transition in glioma cells (Wang et al., 2017). Our results showed that HDAC1 was highly expressed in glioma tumor tissue and significantly correlated with patient survival and immune infiltrate density. Moreover, *HDAC1* had the highest mRNA expression level of all HDACs in 12 glioma cell lines. A Cox proportional hazard model of HDACs and clinical factors disclosed that *HDAC1* expression was significantly correlated with clinical outcome in LGG. *HDAC1* upregulation was significantly correlated with poor OS and DFS in patients with glioma patients. The foregoing associations merit further investigation. We plan to knock out or use the expression of protease inhibitor *HDAC1* in glioma cell lines to observe tumor cell invasion and migration in functional experiments. Besides use tumor-bearing nude mice to further verify whether its *HDAC1* can inhibit tumor growth.

Recent observations demonstrated that class I HDACs (HDAC1, HDAC2, and HDAC3) modulate DNA damage signaling (Miller et al., 2010; Thurn et al., 2013), maintain genomic stability, and prevent tumorigenesis *in vivo* (Dovey et al., 2013; Heideman et al., 2013; Santoro et al., 2013). The mammalian class I deacetylase HDAC2 has been extensively studied. HDAC2 downregulation markedly inhibits tumor growth. Thus, HDAC2 may be an oncogene in tumorigenesis (Zhu et al., 2004). HDAC2 protein overexpression was detected in human gastric, prostate, and breast cancers (Nakagawa et al., 2007; Jinwon et al., 2014). HDAC2 represses gene expression by deacetylating H4K16ac (Ma et al., 2013), determines transcription repression, and participates in the nucleosome remodeling deacetylase complex. *In vitro* and *in vivo* experiments demonstrated that silencing TRPS1 inhibited tumor growth, whereas HDAC2 overexpression promoted it (Wang et al., 2018). The results of the present study corroborated these findings. *HDAC2* expression was higher in glioma tumor than in normal tissues. *HDAC2* expression was also significantly correlated with glioma grade and positively correlated with purity infiltration. However, we found that unlike mRNA expression, immune combination expression was elevated in both glioma and normal tissues possibly because of gene regulation during transcription and translation.

During thymocyte development, HDAC3 is required for positive selection and CD4 lineage development (Stengel et al., 2015). HDAC3 represses CD8 lineage genes to keep double-positive thymocytes in a bipotential state (Philips et al., 2019). Here, *HDAC3* expression in LGG was positively correlated with the infiltration of B cells, neutrophils, and dendritic cells. *HDAC3* mRNA expression was significantly associated with clinical outcome in GBM. Hence, immunity-related cells may be implicated in glioma pathogenesis.

HDAC4 is a key member of class IIa HDACs and performs a wide variety of functions (Seto and Yoshida, 2014). In various cells, HDAC4 is post-transcriptionally regulated by several microRNAs such as miR-1, miR-29, miR-140, miR-155, miR-200a, miR-206, and miR-365 (Wang et al., 2014). Some studies have shown that the molecular mechanism of glioma formation involved HDAC4-mediated SP1 and KLF5 deacetylation in selective MKK7 transcription and oncogenic JNK/c-Jun cascade activation. These findings were consistent with the findings of our research. The results of the immune combination showed that HDAC4 is highly expressed in glioma tumor tissues. KEGG enrichment analysis has revealed that in cancers, HDAC and its neighboring 50 genes are enriched in microRNAs. *HDAC4* upregulation was significantly correlated with poor OS and DFS in all patients with glioma. A Cox proportional hazard model of HDAC and its clinical factors disclosed that *HDAC4* expression was significantly correlated with clinical GBM outcome.

HDAC5 is a class II HDAC. Aberrant HDAC5 expression has been observed in multiple cancer types. HDAC5 participates in cell proliferation and invasion, immune response, and maintenance of stemness (Yang et al., 2021). In the present study, *HDAC5* expression was low in tumor cell lines and in glioma and normal tissues. However, *HDAC5* upregulation was significantly correlated with tumor stage, poor OS, and DFS in all patients with glioma.

HDAC6 and 10 are class IIb HDACs based on their subcellular localizations and expression patterns (Haberland et al., 2009). Previous research has shown that HDAC6-selective inhibitors delayed tumor initiation and progression *in vivo* without causing any significant adverse effects (Auzmendi-Iriarte et al., 2019). HDAC6 inhibition induces an *in vivo* delay in tumor growth and downregulates PD‐L1 expression. Several key immunological checkpoint modulators are regulated by HDAC6 (Lienlaf et al., 2016). *HDAC10* expression is positively associated with PD-L1 expression and may predict the outcome of patients with non-small cell lung carcinoma. HDAC10 inhibition combined with doxorubicin administration kills neuroblastoma but not non-malignant cells by impeding drug efflux and enhancing DNA damage. This novel mechanism targets chemotherapy resistance (Johannes et al., 2018). In this study, *HDAC6* expression was higher in glioma tumor than in normal tissues. Low *HDAC6* mRNA expression was significantly correlated with OS and DFS in patients with glioma. *HDAC10* expression is moderate in LGG and elevated in high-grade gliomas. However, low *HDAC10* mRNA expression was not significantly correlated with DFS in patients with glioma.

HDAC7 sustains human mammary epithelial cell proliferation and favors the establishment of stem‐like cell populations by maintaining a proficient microenvironment. High HDAC8 expression levels in human GBM tissues and GBM-R cell lines were correlated with *O*-methylguanine-DNA methyltransferase levels (Cutano et al., 2019). An inhibitor of HDAC8 combined with temozolomide administration induced WT-p53-mediated apoptosis via WT-p53-mediated *O*-methylguanine-DNA methyltransferase inhibition in GBM-R cell lines (Tsai et al., 2021). Elevated HDAC9 expression is associated with poor prognosis and promotes malignancy (Huang et al., 2018; Linares et al., 2019; Li et al., 2020). *HDAC9* deficiency promoted tumor progression by decreasing CD8^+^ dendritic cell (DC) infiltration in the tumor microenvironment. *HDAC9* expression was significantly positively correlated with CD8^+^ cell counts in human lung cancer stroma samples (Ning et al., 2020). In the present study, immune cell infiltration was associated with HDACs in glioma. *HDAC9* expression in LGG was positively correlated with CD8^+^ cell infiltration. *HDAC7* expression in LGG was positively correlated with the infiltration of CD4^+^ T cells, macrophages, and dendritic cells. *HDAC8* expression in LGG and GBM was positively correlated with purity infiltration. *High HDAC7* mRNA expression was significantly correlated with OS and DFS in patients with glioma. However, *HDAC8* mRNA expression levels were not meaningful for prognostic survival analyses of patients with glioma. *HDAC9* mRNA expression levels were not associated with OS in patients with glioma.

HDAC11 is the only member of the class IV HDACs (Seto and Yoshida, 2014). There is limited information regarding its functions. Using a novel class of highly selective HDAC inhibitors and genetically deficient mouse models, the foregoing study revealed that HDAC11 was necessary for oncogenic JAK2-driven myeloproliferative neoplasm cell and tissue proliferation and survival (Yue et al., 2020). Other data demonstrated a significant role of HDAC11 in mitotic cell cycle progression and survival of MYCN-amplified neuroblastoma cells and suggested that HDAC11 is potentially a valuable drug target (Thole et al., 2017). The present study arrived at the same conclusions. KEGG enrichment analysis disclosed that enrichment of HDAC and its neighboring 50 genes was associated with the cell cycle. *HDAC11* expression was lower in glioma tumor than in normal tissues. *HDAC11* downregulation was significantly correlated with tumor stage, poor OS, and DFS in patients with glioma.

There are several limitations to our study. Firstly, we only analyzed data retrieved from online databases, we must validate our findings using large cohorts. Secondly, our HDAC IHC and immunofluorescence data were incomplete. Third, we did not use our cell lines or tissues to further verify the results. Although HDAC family expression data are similar for glioma cell lines and pancancer analyses. Perhaps, Basic experiments must be conducted to elucidate the mechanisms of HDAC family members and determine their impact on epigenetics. At present, a large number of HDAC inhibitors have been used in other tumors, and we can use HDAC inhibitors to act on cells or animals to further observe their inhibition of tumors. The conventional histone acetylases show lack of blood-brain barrier permeability in the treatment of brain diseases, but through the development of new small molecule probes and carriers, they can penetrate the blood-brain barrier well. we may use nano-encapsulation to further break through the blood-brain barrier (Dang, 2014). In the actual human clinical safety and effectiveness test, we can observe the immunohistochemical expression of HDAC in clinical work and combine with traditional glioma gene diagnosis to analyze and judge the prognosis of glioma diseases.

## Conclusion

Most of these histone Acetylases are usually adjunctive to other chemoradiotherapy drugs such as temozolomide/bevacizumab. However, most of the current clinical trials are phase I/II clinical trials, and further phase III trials are needed to demonstrate the role of histone acetylases in gliomas. In a phase two Study of Concurrent Radiation Therapy, Temozolomide, and the Histone Deacetylase Inhibitor Valproic Acid for Patients With Glioblastoma, histone inhibitors may improve outcomes for patients with glioma compared with historical data (Krave, 2015). In the present study, we systematically analyzed HDAC expression and prognostic value in glioma and revealed the heterogeneity and complexity of the molecular mechanisms associated with these cancers. We also investigated the mRNA expression patterns, prognostic values, genetic alterations, GO enrichment, and PPI networks of HDACs in patients with glioma. According to the immune prognosis analysis, the age and *HDAC1* mRNA expression of patients with LGG were related to disease prognosis. Patient age, CD4^+^ T cell density, and *HDAC3* and *HDAC4* mRNA expression were associated with high-grade glioma prognosis. *HDAC1* and *HDAC2* were relatively upregulated whereas *HDAC11* was downregulated in LGG and GBM tumor tissues. *High HDCA1* and low *HDAC11* mRNA expression levels were significantly related to glioma OS and DFS. HDAC2 mRNA upregulation had prognostic value in OS. The foregoing results indicate that HDAC1/2 are potential prognostic biomarkers for patients with glioma. The present study may facilitate the discovery of novel prognostic biomarkers for glioma among other HDAC family members.

## Data Availability

The original contributions presented in the study are included in the article/Supplementary Materials, further inquiries can be directed to the corresponding authors.

## Abbreviations

CNS, central nervous system; GBM, glioblastoma; IHC, immunohistochemistry; HDAC, histone deacetylase; GEPIA, gene expression profiling interactive analysis; TCGA, The Cancer Genome Atlas; CCLE, Cancer Cell Line Encyclopedia; KEGG, Kyoto Encyclopedia of Genes and Genomes; LGG, lower-grade glioma; TIMER, Tumor Immune Estimation Resource.
